# Central Odontogenic Fibroma of Simple Type

**DOI:** 10.1155/2014/642905

**Published:** 2014-11-23

**Authors:** Prasanth Thankappan, Naga Sirisha V. Chundru, Rajesh Amudala, Prashanthi Yanadi, S. A. K. Uroof Rahamthullah, Meeramma Botu

**Affiliations:** ^1^Department of Oral and Maxillofacial Pathology, CKS Theja Institute of Dental Sciences and Research, Chadalawada Nagar, Renigunta Road, Tirupati, Andhra Pradesh 517501, India; ^2^Department of Oral and Maxillofacial Surgery, CKS Theja Institute of Dental Sciences and Research, Chadalawada Nagar, Renigunta Road, Tirupati, Andhra Pradesh 517501, India

## Abstract

Central odontogenic fibroma (COF) is an extremely rare benign tumor that accounts for 0.1% of all odontogenic tumors. It is a lesion associated with the crown of an unerupted tooth resembling dentigerous cyst. In this report, a 10-year-old male patient is presented, who was diagnosed with central odontogenic fibroma of simple type from clinical, radiological, and histopathological findings.

## 1. Introduction

The 1992 WHO classification defines a central odontogenic fibroma (COF) as “a fibroblastic neoplasm containing varying amounts of apparently inactive odontogenic epithelium” [1]. It is considered to be derived from mesenchymal tissue of dental origin such as periodontal ligament, dental papilla, or dental follicle [2].

Radiographically, COF usually appears as a unilocular radiolucency with well-defined borders but may also exhibit a multilocular appearance with scalloped margins [3]. The tumor sometimes produces an expansile multilocular radiolucency similar to that of the ameloblastoma. Rarity of this tumor often excludes it from most differential diagnosis lists and when diagnosed, it is mostly an unexpected finding that usually requires expert second opinion for confirmation. Most radiographic presentation will suggest the more common radiolucent odontogenic cysts and tumors such as an odontogenic keratocyst, ameloblastoma, and odontogenic myxoma as well as ameloblastic fibroma in children and teenagers. In younger individuals the presentation will also suggest a central giant cell tumor.

## 2. Case Report

A 10-year-old male patient presented with a painless swelling in the left side of the lower jaw of six-month duration which had gradually increased in size.

Extra oral examination revealed facial asymmetry with a hard swelling on the left side of the lower jaw. Intraoral examination revealed swelling extending from distal aspect of the 36 to retromolar area. There was no evidence of paresthesia. On palpation, swelling was firm in consistency.

**Figure 1:**
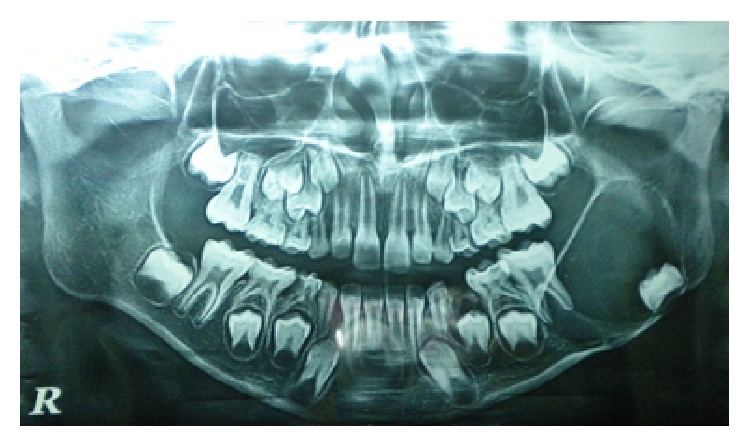
OPG showing well-defined unilocular radiolucency on the left side of the mandible.

Orthopantomogram (OPG) revealed a large well-defined unilocular radiolucent lesion surrounded by sclerotic border on left side of mandible extending anteriorly from developing 35 to half of the ramus posteriorly and superiorly close to coronoid process to inferiorly lower border of mandible. Developing 37 was pushed to the lower end of the radiolucent area (Figure 1).

The differential diagnosis at this stage included dentigerous cyst, odontogenic keratocyst, and ameloblastoma. An incisional biopsy of the lesion was performed for the histopathological examination.

Gross examination of the biopsy specimen showed several soft tissue bits measuring about 1 × 1 cm in size, whitish in color and firm in consistency.

**Figure 2:**
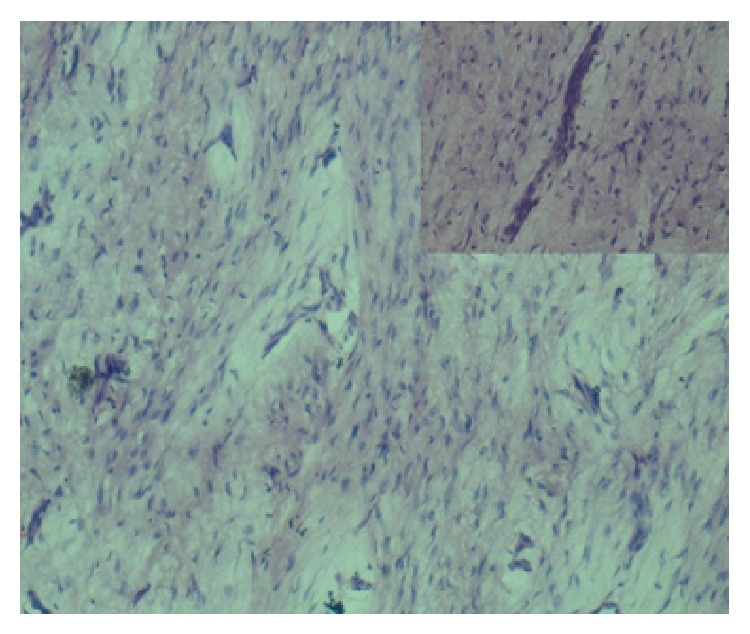
Odontogenic epithelial islands in the form of nests (10x). Inset showing epithelial islands in the form of long strands (40x).

Microscopic examination revealed tumor composed of inactive appearing odontogenic epithelial islands in the form of long strands or isolated nests set in a background of myxoid to cellular connective tissue stroma. Connective tissue also showed fine fibrocollagenous matrix (Figure 2).

Based on clinical, radiographic, and histopathological findings, a diagnosis of central odontogenic fibroma of simple type was established. The patient was then subjected to enucleation of the lesion and had been followed up for one year postoperatively and there was no recurrence noticed.

## 3. Discussion

COF is an extremely rare benign neoplasm that is most often found in females and the incidence between maxilla and mandible is 1 : 1 [4]. It is found in all ages ranging from 11 to 80 years, with an average patient age of 29 years [5].

Radiographically it is mostly unilocular, but, however, larger lesions show multilocular radiolucency with well-defined borders, sometimes with radiopaque areas noted in the interior of the lesion.

Multilocular lesions usually show more aggressive behavior with complications of resorption of teeth, radicular displacement of adjacent teeth, and expansion of cortical bones.

Majority of COFs are clinically asymptomatic causing slow expansion of the cortical bones. Clinical signs often observed are prominence of vestibular cortical and lingual bone (75%), pain (50%), and rhizolysis (interruption of spinal nerve roots by coagulation with radiofrequency waves) (37.5%) [6]. Our case occurred in 10-year-old male patient in mandible and manifested as asymptomatic swelling.

Clinical differential diagnosis includes cysts of odontogenic origin, ameloblastoma, adenomatoid odontogenic tumor, and ameloblastic fibroma. The correct diagnosis is often arrived after histological examination of the lesion [7].

According to the latest classification of odontogenic tumors reported by Gardner, the odontogenic fibroma is classified as a benign lesion derived from “odontogenic ectomesenchyme with or without odontogenic epithelium.” He also has referred the tumor made up of connective tissue and odontogenic islands resembling dental follicle as the simple type and to the tumor described by the WHO as the WHO-type COF [8].

The current classification of odontogenic fibroma by WHO (2005) is (1) the WHO variant, and (2) the non-WHO variant. The WHO variant is considered as a mesenchymal odontogenic tumor and is comprised of two distinct cell types, a fibrous element, and an epithelial component that resembles dental lamina or its remnants. In contrast, the non-WHO variant lacks an epithelial component and is said to be a monomorphic fibroblastic tumor, purported to be of odontogenic mesenchymal origin and ostensibly derived from pulpal or follicular fibroblasts [9].

Histologically the simple type is characterized by a tumor mass made up of mature collagen fibers interspersed usually by many plump fibroblasts that are very uniform in their placement and tend to be equidistant from each other. Small nests or islands of odontogenic epithelium that appear entirely inactive are present in variable but usually in quite minimal amounts. WHO type consists of relatively mature but quite cellular fibrous connective tissue with few to many islands of odontogenic epithelium. Osteoid, dysplastic dentin, or cementum-like material is also variably present. Our case resembled the Simple type.

The histological differential diagnosis of COF includes ameloblastic fibroma (AF), desmoplastic fibroma (DF), and myxoma. AF is composed of epithelial component in the form of strands and islands, showing peripheral layer of cuboidal or columnar cells, which may enclose a small number of cells resembling stellate reticulum, and connective tissue component in AF is more cellular and embryonic looking. DF on the other hand shows aggressive behavior and is devoid of epithelial component and the fibroblasts are myofibroblastic in nature [10]. The presence of epithelial islands is a prerequisite for the diagnosis of COF, whereas it is not a frequent finding in odontogenic myxoma [9].

The treatment of COF is conservative surgery by the enucleation of the lesion. Recurrence is not common. Causes of recurrence are not related to histologic type but due to an incomplete surgical removal of the lesion [11].

## 4. Conclusion

The purpose of presenting this case is to highlight the importance of histopathological examination of every tissue submitted for arriving at confirmatory diagnosis in addition to clinical and radiographic findings.
